# Social Consanguinity, near Kin, Early and Late Marriage

**Published:** 1901-05

**Authors:** Eugene S. Talbot


### SOCIAL CONSANGUINITY, NEAR KIN, EARLY AND LATE MARRIAGE.[1]

BY EUGENE S. TALBOT, M.D., D.D.S.[2]

From the general principles of heredity elsewhere laid down,[3] it must be obvious that the influence of intermarriage in families has been over-estimated as a factor *per se* in producing defect. The idea of the advantages of cross-breeding which seemingly appeared in the practice of exogamy (marriage outside the tribe, or more often outside those having the same totem or coat of arms) arose,

---

[1] Advance chapter from the fourth edition of "Irregularities of the Teeth."

[2] Fellow of the Chicago Academy of Medicine.

[3] Chapter II. of the work.

as I have elsewhere shown, from observation of deformities [1] following intermarriages contracted after the killing of girls for economic reasons has led to exogamy. The idea of incest [2] was of religious origin rather than innate, since totemic relationship (which was chiefly prohibited) was far from being consanguineous. The totem was a mark indicating descent from a supposed animal ancestor endowed with occult powers. The children with the bear totem of one tribe could not marry those having the bear totem of any other tribe. From this practice sprang the medical, theologic, and legal notions anent the danger from marriages of consanguinity, which, as D. Hack Tuke [3] remarks, insisted upon from time to time by medical writers, has been recognized by ecclesiastic authority, civil law, and by popular feeling. By ecclesiastic and civil law, marriage of those very nearly related has been forbidden on other grounds than that of alleged danger to offspring. At the same time the justice of such laws receives support from medical observations, which tend to show that intermarriage may produce degeneracy, idiocy, and insanity. I have elsewhere analyzed this evidence,[1] and shown that for the facts there is more than one explanation. The explanation pointed out by Strahan [4] underlies the chief danger in intermarriages. With a perfectly healthy stock, as every breeder of animals knows, remarks Strahan, "in-and-in breeding" may be practised with impunity, but where the stock is tainted with disease or imperfection, safety is only to be found in "crossing."

The error of the old doctrine upon which was founded the prohibition of consanguineous unions lay, as Strahan remarks, not in asserting that disease and deformity were more met with in children of these than those of other unions, for such is the fact, but in attributing these unhappy results merely to parental blood kindred. Over and above the fact that these consanguineous marriages are almost certain to transmit, in an accentuated form, defect or tendency to disease already present in the family, there is no physiologic reason why such marriages should not take place. Breeders of prize stock frequently breed "in-and-in," not only with impunity, but with marked benefit. But this fact, while going

[1] Degeneracy.

[2] Durkheim Annêe Sociologique, 1898.

[3] Psychological Dictionary.

[4] Marriage and Disease.

to prove that it is not the mere blood relationship of the parents which induces the degeneration too often found in the children of consanguineous marriages, can but rarely be advanced as an argument in support of the marriage of blood relations. The stock-raiser only permits the more perfect members of his flock and herds to continue their kind. For this reason "in-and-in" breeding is innocuous, just as it would be in the human family under like conditions.

Recently acquired characters, whether physiologic or pathologic, are very liable to disappear when the individual bearing such characters intermarries with another not having the same character. The natural tendency in all such cases is to revert in the offspring to the normal or healthy type, so that, unless the new character be very deeply impressed upon the parental organism, it is almost certain it will not appear in the offspring if the other parent have nothing of the character. But when both parents are possessed of the character, whether it be physiologic or pathologic, this natural tendency to revert to the original is often overborne and the character is repeated in an accentuated form in the offspring.

Now, this accentuation of family characters is what must always happen in the case of consanguineous marriages. If there be taint in the family, each member will have inherited more or less of it from the common ancestor. Take the case of cousins the descendants of a common grandparent who was insane and of an insane stock. Here the cousins are certain to have inherited more or less of the insane diathesis. Even if the taint have been largely diluted in their case by the wise or more likely fortunate marriages of their blood-related parents, yet will they have inherited a certain tendency to nervous disease, and if they marry they must not be surprised if that taint appear in aggravated form in their children. Some children of such parents are idiotic, epileptic, dumb, or lymphatic, and the parents marvel whence came the imperfection. In some cases the parents, and possibly grandparents, of the unfortunate children have not displayed any obvious evidence of the tendency to disease which they have inherited and handed on to their descendants. Not looking farther back, the parents boldly assert that such a thing as insanity, epilepsy, scrofula, etc., is unknown to their family. They themselves have never been insane; why, then, should their children be? In like manner children may be epileptic, blind, deaf-mute, lymphatic, cancerous, criminal, drunkards, or deformed from direct inheritance, and yet the family line be honestly declared to be healthy.[1]

The truth of Sir William Aitken's maxim, that "a family history including less than three generations is useless and may even be misleading," is hence obvious. Similarity of temperament induced by a common environment, which Strahan calls "social consanguinity," is hence also a potent factor in the production of all hereditary degenerations. Living under similar customs, habits, and surroundings, laboring at the same occupation, indulging in the same dissipation, tend to engender like diseases and degenerations irrespective of any blood relationship. Hence it not seldom happens that persons not even distinctly related by blood are in reality much more nearly related in temperament than cousins or even nearer blood relations who have experienced widely different modes of life. This "social consanguinity" is the great curse which dogs every exclusive tribe and class and hurries them to extinction. It has largely aided real or family consanguinity in the production of the diseases and degenerations which have so heavily fallen upon the aristocracies and royal families of Europe. This "social consanguinity" appears likewise in the tendency of the neurotic to intermarry, popularly expressed in the proverb that "like clings to like." This likeness in mental characteristics has been shown to be present by Roler, de Monteyel, Kiernan, Bannister, and Manning, so far as Germany, France, the United States, and Australia are concerned. Bannister puts the statistic proof of this tendency thus forcibly: "There are in Illinois, according to the most recent estimates in round numbers, about six thousand insane, or one to a little over five hundred of the population. Even if we double, treble, or quadruple this frequency, to include all that have been or are to be insane, as well as those insane at the present time, it would not appear that there was much probability of two insane persons being married according to any ordinary law of chances.

---

[1] Heredity consists in the transmission of sufficient hereditary potency to pass the inherited quality through intra- and extrauterine periods of stress when certain functions and organs disappear to give way to others developing. At one of these periods the polyphyodont tendency gives way to the diphyodont. Through the influences of these periods of stress marked acquired defect becomes minor or *vice versa.* Through the period of stress the influence of benign or malign atavism is exerted. See Chapter II. of the fourth edition of Talbot's "Irregularities of the Teeth."

In fact, we find that in four out of the one hundred and four with insane heredity both father and mother had been insane. In one of these cases the insane heredity involved both parents and both grandparents on each side, though in the case of the latter the histories show it only as collateral. In the cases of the three patients with direct paternal and collateral maternal heredity, two had also direct maternal and collateral paternal heredity, and in one case there was collateral heredity of insanity on both sides. This makes altogether ten per cent. of those with insane heredity, with it on both sides, maternal and paternal, and thus favored with a double opportunity to inherit mental disease. If to this be added the instances where with insanity of one parent there is either epilepsy, hysteria, drunkenness, brain disease, nervousness, etc., of the other, the ratio of double inheritance rises to over twenty per cent."

Since jaws and face are transitory structures, but relatively little taint is needed in a family or community to cause suppressive or hypertrophic degeneration of the face and jaws and irregularities of the teeth. The influence of this factor of these neurotic and "social consanguinity" tendencies in the production of deformities of the face and jaws and irregularities of the teeth cannot well be over-estimated. A test of these deformities is alleged to exist in the Polynesian populations of the Pacific Islands, where race admixture can be excluded for a relatively long period. Concerning the ancient Hawaiians, J. M. Whitney [1] remarks: "Here is a people isolated from all others for at least fourteen hundred years, with no admixture of races, yet irregularity of the teeth of both maxillæ is almost as common as it is among the mixed races of to-day." In social consanguinity of the Polynesians must be peculiarly reckoned their excessive and systematized licentiousness, shown in societies for the practice of extreme sexual indulgence like the Areoi.[2] Such societies would undoubtedly create neurotic states and tendencies and produce more marked degeneracy of the face, jaws, and teeth than intermixture of race or consanguineous marriage. The factors of race admixture cannot, as Denniker [3] has shown, be completely excluded from consideration among the ancient Hawaiians. Since leprosy, like syphilis, may

---

[1] The World's Columbian Dental Congress Transactions, page 109.

[2] Schultze: Fetichism.

[3] Races of Men.

simply check development without causing infection *in utero,* this factor has likewise to be taken into consideration. Furthermore, as Alvarez,[1] of Wailu, Dahu has shown that mortality among Hawaiian babies is very large. Hygiene is practically unknown to the mothers. Kava-kava (the fermented juice of the awa) is the great medicinal agent of the Kahuma (sorcerer, medicine-man), who is the chief medical resource of the natives. Syphilis is very common, especially the non-venereal type. The habits of the natives aid the spread of the disease. Under such conditions irregularities must result.

Among the factors interfering with the proper development of the child is the condition of the mother at the time of impregnation and during pregnancy. Age at pregnancy, exposure to improper diet, to narcotics, and to the toxic occupations, all play a part in checking fœtal development. So, likewise, do frequently repeated pregnancies. The age of the mother at pregnancy is too much ignored in dealing with defects. J. Mathews Duncan[2] pointed out nearly two decades ago that the offspring of early and senile marriages were defective. Multiple and too nearly repeated pregnancies were of frequent occurrence. Conger[3] (whose results were later corroborated by Joseph Workman[4] and Kiernan[4]) points out that in all degenerative forms age of the parent must be taken into consideration, since it alone often determines degeneracy. Conger found that the age of the mothers of degenerates is below twenty-five years. Korosi,[5] in an investigation of the influence of the age of parents on the vitality of children, found that the proportion of deaths among children from unhealthy constitutions or maladies traceable to the mother was twice as large among the children of mothers under twenty as among the children of mothers over thirty. The healthiest offspring were born of mothers between twenty and thirty united to husbands between thirty and forty. Where either husband or wife was under twenty, the offspring usually proved weakly. This is particularly the case even in Hungary, where the girls become women at thirteen. In that

---

[1] Pacific Medical Journal, 1893.

[2] Sterility, Fecundity, and Allied Topics.

[3] Il Manicomo, May, 1886.

[4] Alienist and Neurologist, 1887.

[5] Orvosi Hetilap, 1894.

country in five per cent. of the number of marriages the brides are under twenty years of age.

Marro[1] finds that among all classes of criminals there is an excess of immature parents (under twenty-five) or senile parents (over forty-two). As I have elsewhere remarked, it is a well-known experience that the children of the aged readily show degenerate types.[2] Arthur Mitchell and Langdon Down have recognized the influence of premature and late marriages in the production of idiocy. Factors capable of producing idiocy may arrest fœtal development at all stages. Kiernan[3] has had under observation a Nova Scotian family of Scotch extraction, the mother of which continued to bear children until she was sixty-three years old. There had been no pregnancy between fifty and fifty-six. At fifty-six a son was born who had ear, jaw, and skull stigmata, and became a periodical lunatic at twenty-five. A son born a year after was a six-fingered idiot with retinitis pigmentosa. Three of the next children were paralytic idiots in infancy. One of the next children was a periodical sexual invert female. The last child was an epileptic. The children born before the age of fifty were normal, and averaged sixty years of age.

